# Phenothiazines alter plasma membrane properties and sensitize cancer cells to injury by inhibiting annexin-mediated repair

**DOI:** 10.1016/j.jbc.2021.101012

**Published:** 2021-07-26

**Authors:** Anne Sofie Busk Heitmann, Ali Asghar Hakami Zanjani, Martin Berg Klenow, Anna Mularski, Stine Lauritzen Sønder, Frederik Wendelboe Lund, Theresa Louise Boye, Catarina Dias, Poul Martin Bendix, Adam Cohen Simonsen, Himanshu Khandelia, Jesper Nylandsted

**Affiliations:** 1 Membrane Integrity, Danish Cancer Society Research Center, Copenhagen, Denmark; 2 PHYLIFE: Physical Life Science, Department of Physics, Chemistry and Pharmacy, University of Southern Denmark, Odense M, Denmark; 3 Niels Bohr Institute, University of Copenhagen, Copenhagen, Denmark; 4 Department of Cellular and Molecular Medicine, Faculty of Health Sciences, University of Copenhagen, Copenhagen, Denmark

**Keywords:** derivatives of phenothiazine, trifluoperazine (TFP), annexin, membrane injury, annexin inhibitor, plasma membrane repair, membrane resealing, membrane curvature, cancer, high intensity focused ultrasound (HIFU), AFM, atomic force microscopy, ANXA, Annexin, MD, molecular dynamics, PE, phosphatidylethanolamine, PM, plasma membrane, PS, phosphatidylserine, TFP, trifluoperazine

## Abstract

Repair of damaged plasma membrane in eukaryotic cells is largely dependent on the binding of annexin repair proteins to phospholipids. Changing the biophysical properties of the plasma membrane may provide means to compromise annexin-mediated repair and sensitize cells to injury. Since, cancer cells experience heightened membrane stress and are more dependent on efficient plasma membrane repair, inhibiting repair may provide approaches to sensitize cancer cells to plasma membrane damage and cell death. Here, we show that derivatives of phenothiazines, which have widespread use in the fields of psychiatry and allergy treatment, strongly sensitize cancer cells to mechanical-, chemical-, and heat-induced injury by inhibiting annexin-mediated plasma membrane repair. Using a combination of cell biology, biophysics, and computer simulations, we show that trifluoperazine acts by thinning the membrane bilayer, making it more fragile and prone to ruptures. Secondly, it decreases annexin binding by compromising the lateral diffusion of phosphatidylserine, inhibiting the ability of annexins to curve and shape membranes, which is essential for their function in plasma membrane repair. Our results reveal a novel avenue to target cancer cells by compromising plasma membrane repair in combination with noninvasive approaches that induce membrane injuries.

The plasma membrane (PM) shapes and protects cells from the extracellular environment. To keep the PM intact and prevent cell death induced by membrane disruptions, eukaryotic cells have developed efficient repair mechanisms to ensure rapid resealing. Repair strategies depend on both membrane fusion and membrane replacement strategies and involve cytoskeletal and endomembrane systems (1, 2, 3). Members of the Annexin (ANXA) protein family (in mammals: ANXA1-11 and ANXA13) are instrumental in coping with PM injuries and are characterized by their Ca^2+^-dependent binding to anionic phospholipids and ability to aggregate vesicles and fuse membranes (4). Injury to the PM induces a prompt recruitment of ANXAs to the damaged membrane, which is triggered by the influx of extracellular Ca^2+^ into the cytoplasm and is achieved by binding to negatively charged phospholipids through their C-terminal core domain (5).

Although the function of ANXAs in repair has merely been attributed to their ability to aggregate and fuse membranes (6), we and others have recently shown that the strong membrane-shaping and curvature-sensing properties of ANXAs are also critical for repair (7, 8, 9). Specifically, ANXA1, ANXA2, and ANXA4-7 are strong inducers of membrane curvature and through a coordinated manipulation of membranes promote PM repair mechanisms (6, 7, 8, 10, 11). For example, recent data from supported membrane models show that ANXA6 induces constriction force, while Ca^2+^-dependent homo-trimerization of ANXA4 at free membrane edges induces curvature force (7). The combined forces, likely in collaboration with other ANXA family members, appear to drive constriction of membrane wound holes in cells by pulling the edges together for eventual fusion (8). Moreover, some ANXAs including ANXA4 and ANXA5 have high affinity for highly curved membranes that appear at free edges near rupture sites, a property that might accelerate their recruitment for rapid repair (12).

With the ubiquitous expression of ANXAs (5) and the frequency of PM injury, the role of ANXAs in mediating PM repair is broadly relevant, but appears to be heightened further in cancer cells (13, 14, 15, 16). Most ANXAs are highly expressed in various cancer cell types (17, 18, 19). This is likely to be related to the increased membrane-associated processes and enhanced membrane stress that cancer cells face (13, 20). The increased metabolic demands of cancer cells (21) drive increased membrane trafficking, a process that relies on the ability of ANXAs to aggregate and fuse membranes (22). The heightened membrane stress has two sources: the increased metabolic activity and the invasive behavior of cancer cells. Firstly, the by-products of increased metabolic activity (oxidative species) may oxidize lipids and proteins at the membrane, resulting in membrane disruptions (23, 24). Secondly, as cancer cells migrate through the basement membrane and the dense interstitial tissue their PM is subjected to tremendous physical stress, causing injuries (25, 26). Thus, inhibiting repair by compromising ANXA function may provide means to sensitize cancer cells to PM damage and cell death.

Derivatives of phenothiazine are heterocyclic cationic compounds that have widespread use, for example, in psychiatry and allergy treatment (27, 28). By intercalating in membrane bilayers and creating lipid phase separation (29, 30), phenothiazines affect membrane properties and can revert multidrug resistance in different types of cancer cells (31). This itself has therapeutic potential, as sensitizing cancer cells, for example, by the phenothiazine derivative trifluoperazine (TFP), may improve the efficacy of cancer chemotherapy (32). Interestingly, phenothiazines including TFP and promethazine were reported to inhibit ANXA2-mediated aggregation and fusion of phosphatidylserine (PS) and phosphatidylethanolamine (PE) containing liposomes *in vitro* (33). Driven by these findings, we hypothesized that phenothiazines can compromise PM repair by inhibiting ANXA function at the membrane. Using a combination of cell biology, biophysics, and simulations (3), we find that phenothiazines, and in particular TFP, strongly sensitize to membrane disruptions by compromising ANXA function. We provide evidence that TFP changes the physical membrane properties, which reduces membrane bilayer thickness, resulting in a membrane that is more fragile and prone to ruptures. Importantly, phenothiazines decrease ANXA binding by inhibiting the lateral diffusion of PS and, in turn, compromise their ability to bend and shape membranes, which is needed for their function during PM repair. This initiates a vicious cycle where membrane thinning sensitizes cells to damage and the compromised ANXA-repair response facilitates membrane injury-induced cell death.

## Results

To investigate if derivatives of phenothiazine can sensitize cancer cells to PM damage, different cancer cell models (Human HeLa cervix carcinoma, NCI-H1299 human non-small-cell lung carcinoma, MCF7, and MDA-MB-231 breast carcinoma cells) were injured by exposure to detergent, heat shock, or mechanical stress by already established membrane injury approaches (8, 11, 34). The sublethal concentration (7–15 μM) was determined for each phenothiazine used (thioridazine, fluphenazine, and TFP) by impermeable propidium iodide exclusion assay (Fig. S1), which were found to be in the same range as previously reported (35, 36, 37). To minimize cell toxicity, cells were incubated with phenothiazines for maximum 4 h (thioridazine and fluphenazine for 30 min and TFP for 4 h).

**Figure 1:**
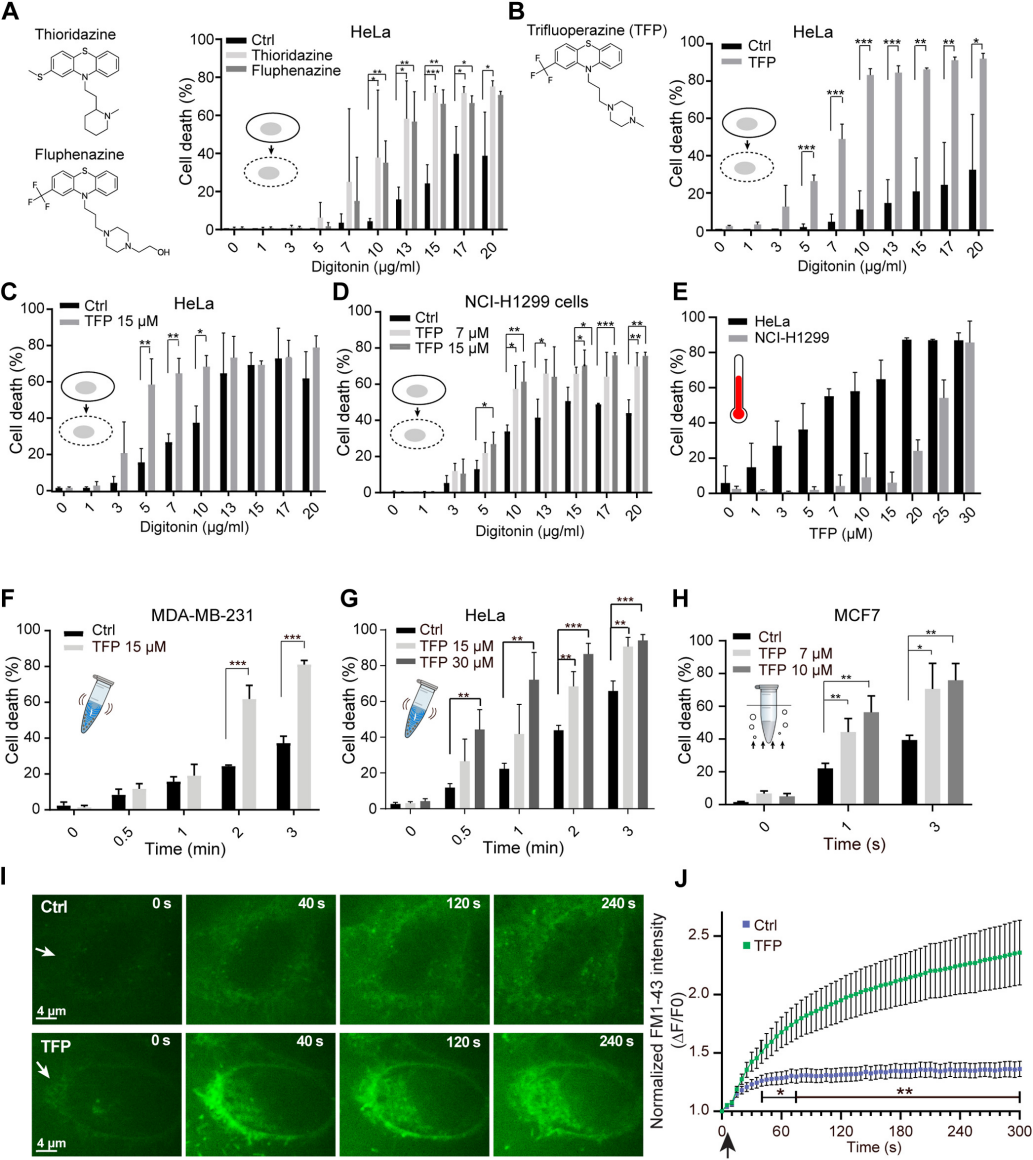
**Phenothiazines sensitize cancer cells to PM injury and cell death triggered by detergent, heat shock, and mechanical stress.***A*, chemical structure of thioridazine and fluphenazine (*left*) and cell death measurements (*right*) of HeLa cells upon digitonin-induced injury (30 min) pretreated with 10 μM thioridazine or 15 μM fluphenazine for 30 min and assayed using cell impermeable propidium iodide and permeable Hoechst-33342. *B*, chemical structure of TFP (*left*) and similar cell death measurements for HeLa cells pretreated with 15 μM TFP for 4 h, or *C*, 15 μM TFP for 30 min, or *D*, NCI-H1299 cells pretreated with 7 or 15 μM TFP for 30 min. *E*, HeLa and NCI-H1299 cells treated either left untreated or pretreated with indicated concentrations of TFP for 45 min and exposed to heat shock for 15 min by increasing temperature from 37 to 55 °C. *F*, MDA-MB-231 cells in suspension pretreated with 30 μM TFP (1 h), or *G*, HeLa cells pretreated with 15 or 30 μM TFP (1 h) and exposed to mechanical injury by vortex with glass beads for 0 to 3 min. *H*, MCF-7 cells pretreated with 7 or 15 μM TFP for 1 h followed by sonication for 1 or 3 s. Error bars represent S.D. for three independent experiments. The *asterisks* represent *p*-values based on a Student's *t* test: ∗*p* < 0.05; ∗∗*p* < 0.01; ∗∗∗*p* < 0.001. *I*, representative sequential images showing FM1-43 dye uptake in HeLa cells treated with 20 μM TFP for 2 h prior to injury by ablation laser (*white arrows* mark site of injury) and *J*, corresponding repair kinetics. Mean ± S.E.M. of quantified and normalized cytoplasmic FM1-43 levels from >9 cells/condition from three experiments. *p*-values based on a Student's *t* test: ∗*p* < 0.05; ∗∗*p* < 0.01.

HeLa cells pretreated with thioridazine and fluphenazine for 30 min showed a significant increase in cell death when injured by a membrane pore-forming detergent, digitonin, for 20 min (Fig. 1*A*) as measured by impermeable propidium iodide exclusion assay. This is also indicative of increased PM permeabilization induced, since propidium iodide is impermeable to uninjured cells. The sensitization effect was more pronounced in cells pretreated with 15 μM TFP for 4 h and subsequently exposed to varying concentrations of digitonin for 30 min, being readily observed at low concentrations of digitonin (from 5 μg/ml) (Fig. 1*B*). Both shorter digitonin treatment (30 min) (Fig. 1, *C* and *D*) and lower TFP concentration (7 μM) (Fig. 1*D*) were capable of sensitizing different cell lines (HeLa and NCI-H1299 cells).

Next, we sought to induce PM injury by other methods: heat stress and mechanical stress. For the former, cells were exposed to increasing temperature (37–55 °C) for 15 min, which increases the thermal motion and rearrangement of membrane phospholipids, inducing small PM disruptions. Varying concentrations of TFP dramatically sensitized cells to heat stress resulting in PM permeabilization and cell death (Fig. 1*E*). For mechanical injuries cells in suspension were mixed with glass beads and injured by vortex or exposed to short bursts of ultrasound by sonication. Pretreatment with TFP followed by glass bead vortexing for 0.5 to 3 min sensitized to PM damage in both MDA-MB-231 (Fig. 1*F*) and HeLa cells (Fig. 1*G*). Furthermore, TFP-treated MCF7 breast carcinoma cells were significantly more vulnerable to membrane disruptions by sonication as compared with untreated cells (Fig. 1*H*). Mild sonication produces pulsed, high-frequency sound waves that disrupt cell membranes (referred to as sonoporation), resulting in cell lysis and the release of cytosolic contents (34).

Lastly, to decipher whether the repair kinetic upon PM injury was altered with phenothiazine treatment, HeLa cells were injured with a UV pulsed laser in the presence of the membrane-impermeable FM1-43 dye and monitored by time-lapse imaging. While control cells were able to repair their PM within 20 to 30 s, as demonstrated by the decline of FM1-43 dye uptake seconds after injury, cells treated with TFP failed to repair and continued to take up the cell impermeable dye (Fig. 1, *I* and *J*). Taken together, phenothiazines (particularly TFP) sensitize cancer cells to PM damage and compromise the repair response.

### TFP compromises ANXA2-mediated PM repair

**Figure 2:**
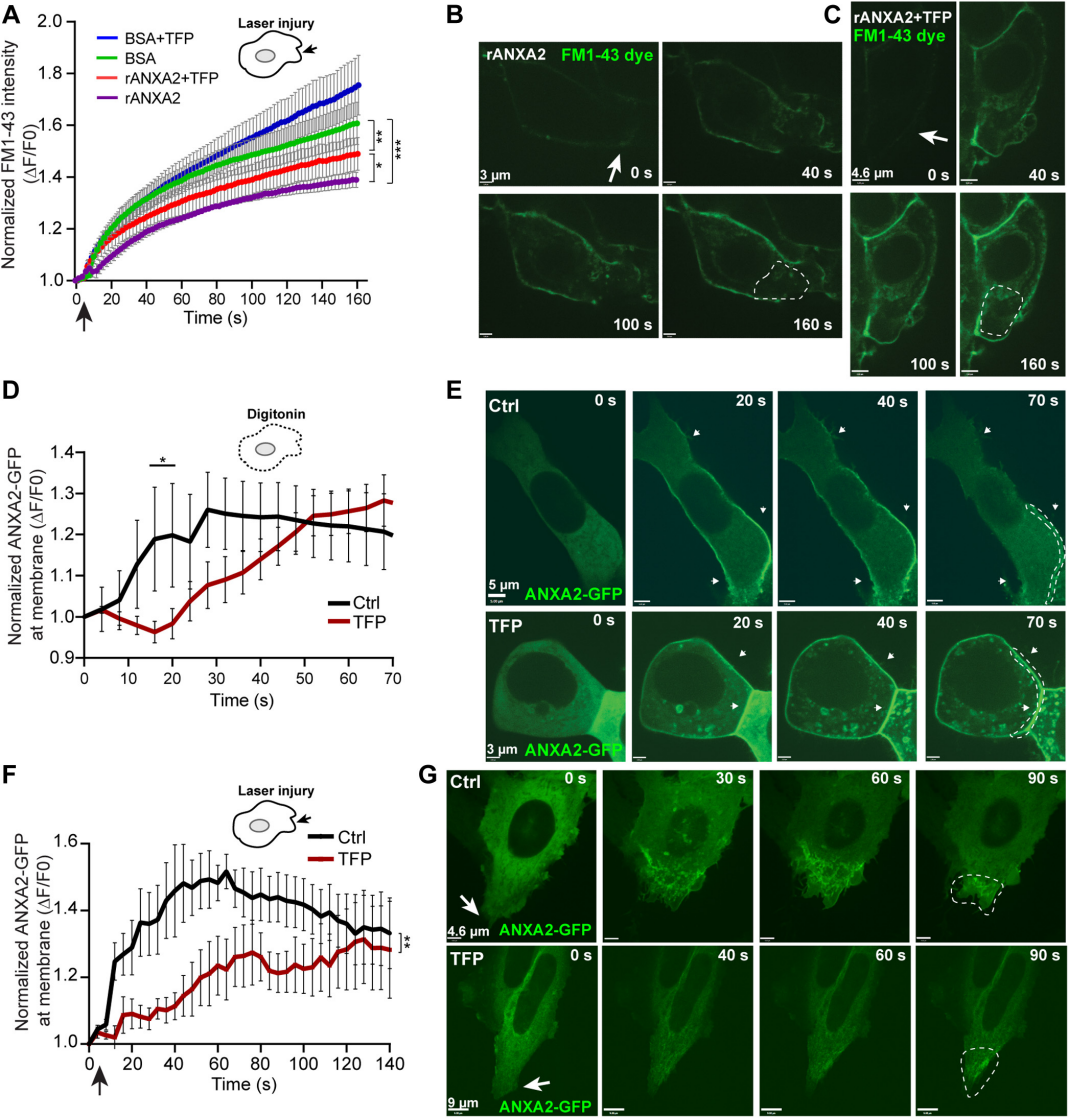
**Trifluoperazine inhibibits PM repair induced by recombinant ANXA2 and compromises ANXA2-GFP translocation to the injured membrane.***A*, recombinant ANXA2 (rANXA2) or bovine serum albumin (BSA) (20 μg/ml) was added to the medium of MCF-7 cells and cells were injured by laser (*black arrow*) in the presence of membrane impermeable FM1-43 dye. Membrane repair kinetic was measured by FM1-43 dye uptake in cells pretreated with rANXA2 and ±3 μM TFP. Means represent ±S.E.M. from ten experiments per condition. Differences were assessed by Kruskal–Wallis test: ∗*p* < 0.05; ∗∗*p* < 0.01; ∗∗∗*p* < 0.001. *B*, representative images showing FM1-43 dye uptake in cells pretreated with rANXA2 or *C*, cells pretreated with rANXA2 + TFP. *Dotted line* indicates area used for quantifying cytoplasmic FM1-43. *D*, translocation behavior of ANXA2-GFP upon digitonin-induced injury (25 μg/ml) in HeLa cells treated with TFP (10 μM for 90 min) as compared with Ctrl. Note that TFP-treated cells show a delay in peak fluorescence intensity at the injured PM. *E*, corresponding live-cell images of ANXA2-GFP transfected cells before and after injury. *White arrows* indicate injured membrane and *dotted line* marks membrane area used for measuring ANXA2-GFP intensity. Mean ± S.E.M. for >17 experiments per condition. Unpaired *t* test with Welch's correction: ∗*p* < 0.05. *F*, translocation behavior of ANXA2-GFP upon laser-injury in HeLa TFP-treated cells (20 μM, 2 h) as compared with Ctrl-treated cells. TFP-treated cells show reduced ANXA2-GFP fluorescence intensity at the injured membrane. *G*, corresponding representative images obtained from time-lapse videos. The *asterisks* represent *p*-values based on a Student's *t* test: ∗*p* < 0.05. Mean ± S.E.M. from eight experiments per condition.

In the light of the reported ability of phenothiazines, including TFP, to inhibit ANXA-mediated aggregation of liposomes *in vitro* (33), we next tested if TFP can compromise ANXA-mediated repair. For this, we added recombinant human ANXA2 to MCF7 cells extracellularly and subjected cells to laser injury, as previously described. The presence of ANXA2 in the medium promoted a significantly improved repair response as compared with controls upon laser injury (Fig. 2, *A* and *B*). However, TFP pretreatment compromised this ANXA2-mediated repair effect, resulting in more FM1-43 dye uptake (Fig. 2, *A*–*C*). To discard differences in basal membrane permeability driven by the effect of TFP in membrane properties, intracellular FM1-43 dye was normalized by the intensity at preinjury. Thus, TFP seems to disturb the repair function of ANXA2.

To address this further, we investigated if TFP directly inhibits the recruitment and accumulation of ANXAs at the damaged membrane after injury. HeLa cells were transfected with ANXA2 coupled to GFP (ANXA2-GFP) and monitored by time-lapse imaging during digitonin treatment or upon laser injury. In cells treated with digitonin, ANXA2-GFP translocation to the PM occurred within 20 to 25 s, whereas accumulation of ANXA2-GFP in TFP-treated cells took 40 to 45 s (Fig. 2, *D* and *E*). Thus, TFP appeared to delay accumulation of ANXA2-GFP at the wounded membrane. When cells were injured by an ablation laser, ANXA2-GFP accumulation peaked at the damaged membrane within 35 to 45 s in contrast to 120 to 130 s in TFP-treated cells (Fig. 2, *F* and *G*). Furthermore, the degree of ANXA2-GFP recruitment to the membrane (*i.e.*, the peak normalised intensity of ANXA2-GFP at the membrane) was significantly reduced in the latter condition, which may negatively influence the repair kinetics (Fig. 2*F*). Taken together, these experiments show that TFP delays the accumulation of ANXA2-GFP at the injured PM, suggesting that phenothiazine derivatives may directly compromise ANXA function.

### ANXA-mediated membrane curvature is inhibited by TFP

**Figure 3:**
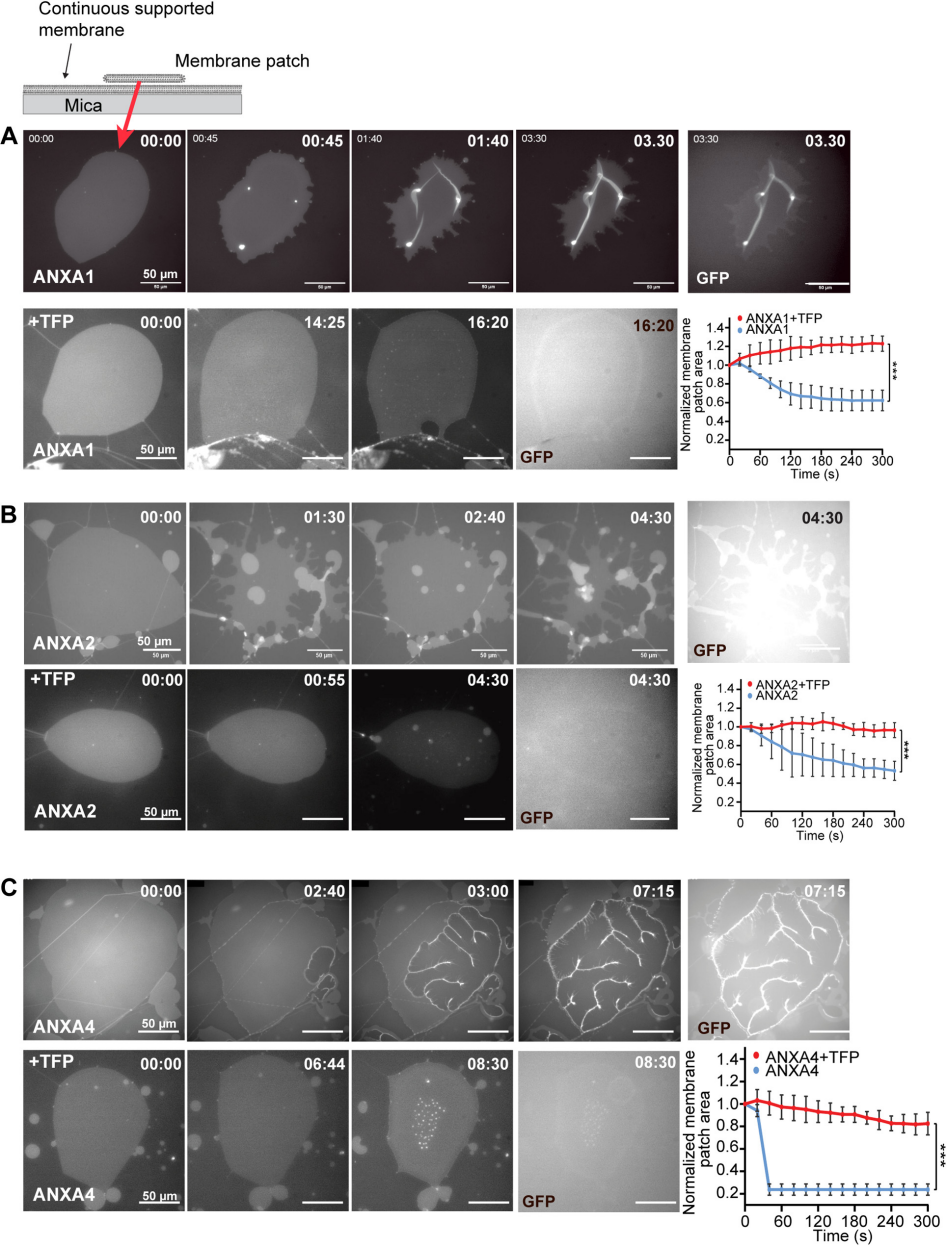
**Membrane curvature induced by ANXA1, ANXA2, and ANXA4 is inhibited by trifluoperazine.** Schematic of supported membrane model composed of nonvesicular membrane patches with open edges (*top*). Response of membrane patches stained with DiD before and after addition of recombinant ANXA-GFP protein ±15 μM TFP in the presence of Ca^2+^. *A*, representative sequential images of a membrane patch with ANXA1-GFP protein (*upper panel*) and ANXA1-GFP protein + TFP (*lower panel*). Last image in rows (GFP channel) reveals the extent of ANXA1-GFP protein binding to membrane patches. Graph (*right*), corresponding plot showing normalized changes in the membrane patch area after addition of ANXA1-GFP ± TFP. t = 0 indicates time of ANXA addition after TFP intercalation in the membrane. Mean ± S.E.M. of three independent experiments per condition. *p*-values based on unpaired *t* test with Welsh’s correction: ∗∗∗*p* < 0.001. *B* and *C*, same as for *A* but with recombinant ANXA2-GFP protein and ANXA4-GFP respectively. ANXA protein concentrations: ANXA1-GFP: 46 nM, ANXA2-GFP: 58 nM and ANXA4-GFP: 27 nM. Time: min and s. See also Videos S1–S4.

To characterize the impact of phenothiazines on ANXA function at the membrane, we took advantage of a membrane model system composed of membrane patches laying on a primary solid-supported membrane formed after hydration of a precursor lipid film (Fig. 3*A*, top schematic). The planar patches allow out-of-plane bending, away from the supported surface and can be used for monitoring shape changes generated by ANXAs, which critically depends on the initial membrane geometry and on the presence of free membrane edges in particular (3, 8). Hence, this system is useful for modeling the PM near the injury site to address the experimental challenge that a membrane hole contains free edges. Addition of recombinant human ANXA1-GFP or ANXA2-GFP proteins to anionic membrane patches (POPC/POPS, 9:1 M ratio) in the presence of Ca^2+^-induced folding or blebbing phenotypes, where most of the original patch area eventually became converted into folds and bleb structures over a timescale of less than 8 min (Fig. 3, *A* and *B*, upper panels and Video S1). We have previously demonstrated that these annexin-induced phenotypes occur independently of GFP/RFP tags (7, 8). The exposure of membrane patches to recombinant ANXA4 induced strong curvature and membrane rolling as initiated from the free edges as previously observed (7, 8) (Fig. 3*C*, upper panel and Video S3). Imaging of the patches in the GFP channel confirmed that folding, blebbing, and rolling were associated with binding of ANXAs to the patch surface where the protein became incorporated into the curved membrane structures as revealed by the strong fluorescence from these regions (Fig. 3, *A*–*C*, upper panel, GFP channel). Interestingly, addition of TFP completely inhibited ANXA-mediated curvature and membrane shaping for ANXA1, ANXA2, and ANXA4 (Fig. 3, *A*–*C*, lower panels and corresponding plots, right. See also Videos S2 and S4). Furthermore, TFP reduced ANXA binding to the membrane patches, but some ANXAs were still able to bind the patches, as visualized through the GFP channel (Fig. 3, *A*–*C*, lower panel, GFP channel).

These data suggest that TFP impacts on the membrane-shaping capacity of ANXAs and, to some extent, compromises ANXA binding to membrane patches.

### TFP enhances membrane fluidity and reduces membrane thickness

**Figure 4:**
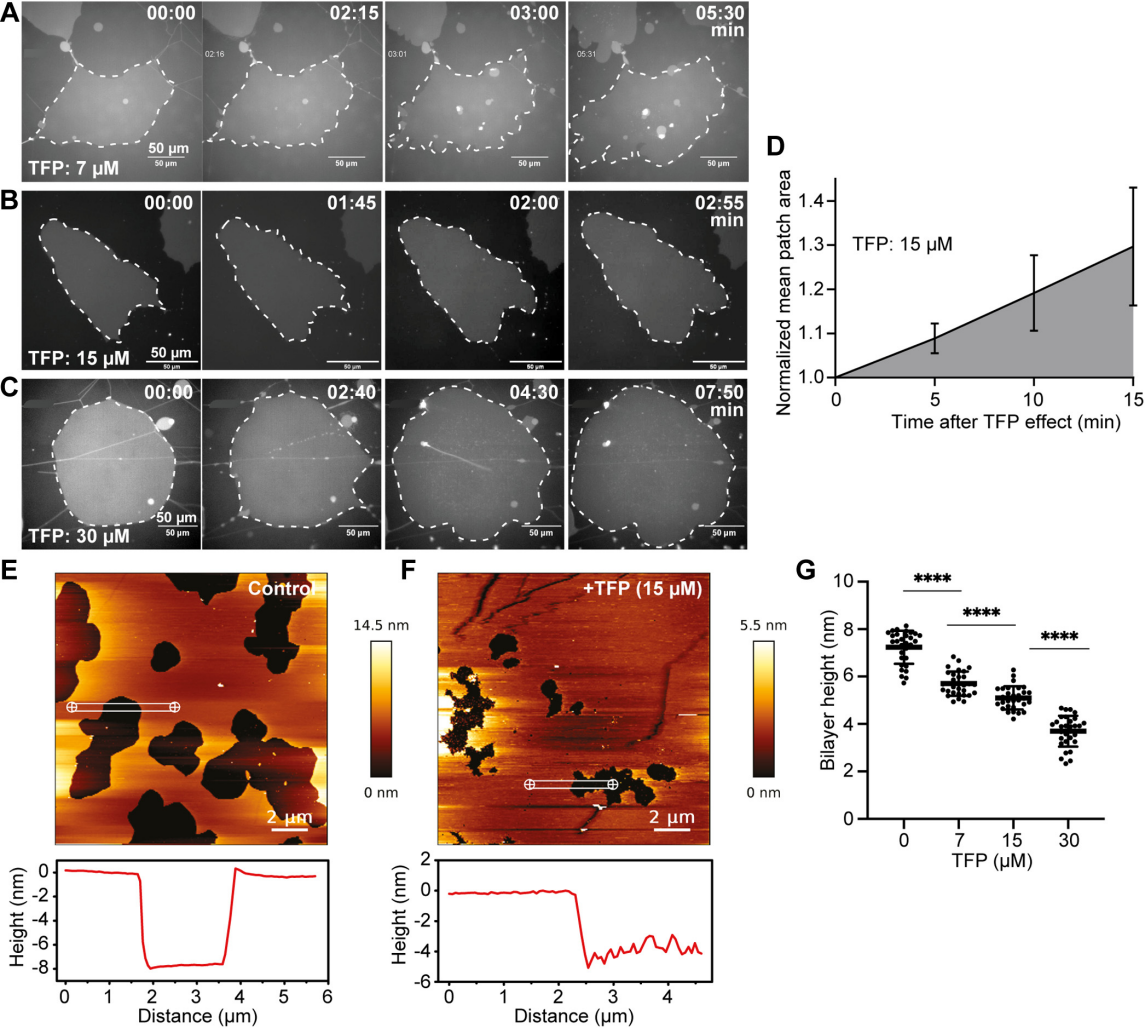
**Trifluoperazine increases membrane surface area and reduces membrane bilayer thickness.***A*–*C*, representative sequential images showing supported membrane patches (stained with DiD) after adding increasing TFP concentrations. *Dotted lines* mark patch areas. *D*, quantification of membrane patch area over time upon adding 15 μM TFP (t = 0 indicates first measurement after TFP intercalation on membrane). Mean ± S.D. of three experiments. *E*, atomic force microscopy (AFM) images of supported lipid bilayers (POPC, POPS (9:1)) in absence and *F*, presence of TFP (15 μM) and corresponding line profiles. *G*, AFM cross-sectional line profiles for determination of bilayer heights for different conditions (0, 7, 15, and 30 μM TFP). Three images of different locations of each sample (one per condition) were taken and ten lines were drawn across hole edges in membrane and used to measure bilayer height (total of 30 line profiles per condition). Unpaired *t* test with Welch's correction was applied to test differences between the conditions: ∗∗∗∗*p* < 0.0001.

To gain further insight into the impact of TFP on the biophysical properties of the membrane, we incubated double-supported membrane patches (POPC/POPS, 9:1 M ratio) with varying concentrations of TFP (7, 15, and 30 μM) and monitored the effect by time-lapse imaging. Here, TFP induced a dose-dependent increase of membrane patch areas over time, suggesting that it intercalates into membranes and affects the packing of lipid molecules (Fig. 4, *A*–*D*). Atomic force microscopy (AFM) was used to scan on the surface of a POPC/POPS (10% PS) bilayer surface. The bilayer, formed by vesicle deposition, was heated to 60 °C and allowed to cool to room temperature. Cooling induced membrane contraction, and holes formed in the bilayer, revealing the mica substrate and allowing for membrane height to be accurately determined before and after successive additions of TFP. In agreement, AFM revealed a significant decrease in membrane bilayer thickness after TFP treatment in a concentration-dependent manner (Fig. 4, *E*–*G*). Taken together, we show that TFP directly increases membrane fluidity and reduces membrane bilayer thickness in accordance with previous studies (38, 39), which may have a drastic impact on binding and membrane-shaping function of ANXAs during repair.

### Molecular dynamics simulations

**Figure 5:**
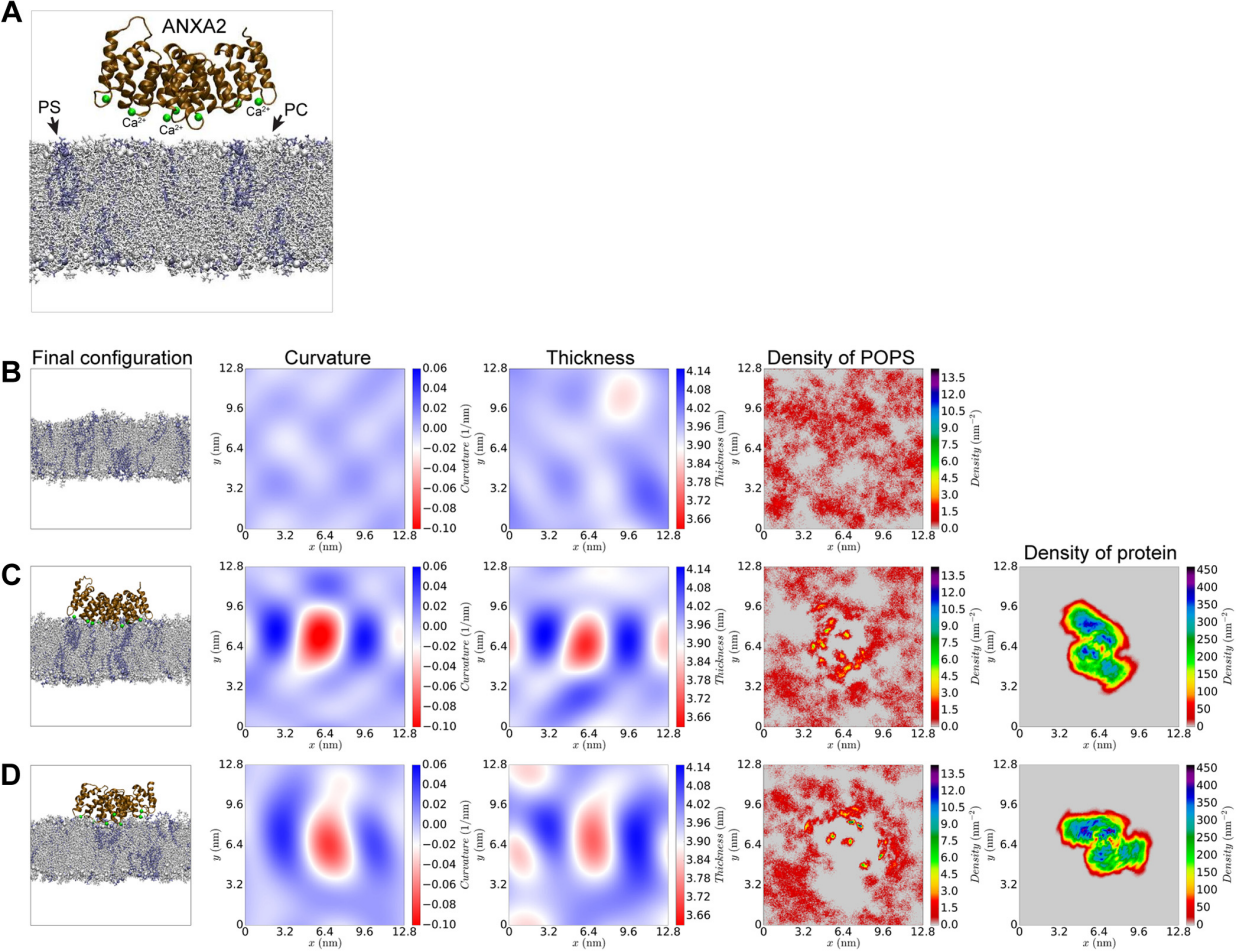
**All atom MD simulations of membrane systems without TFP.***A*, membrane (20% POPS, 80% POPC) + ANXA2 protein. In configuration snapshots, POPC lipids are shown in *gray* and POPS lipids are shown in *blue*. Ca^2+^ ions are shown as *green spheres*. *B*, final configuration of pure membrane and profiles of curvature, thickness, upper monolayer POPS density in systems without TFP molecules. *C* and *D*, two representative simulation replicates of the systems containing ANXA2. See also Video S5 and Fig. S2 for data from the other simulation replicates.

**Figure 6:**
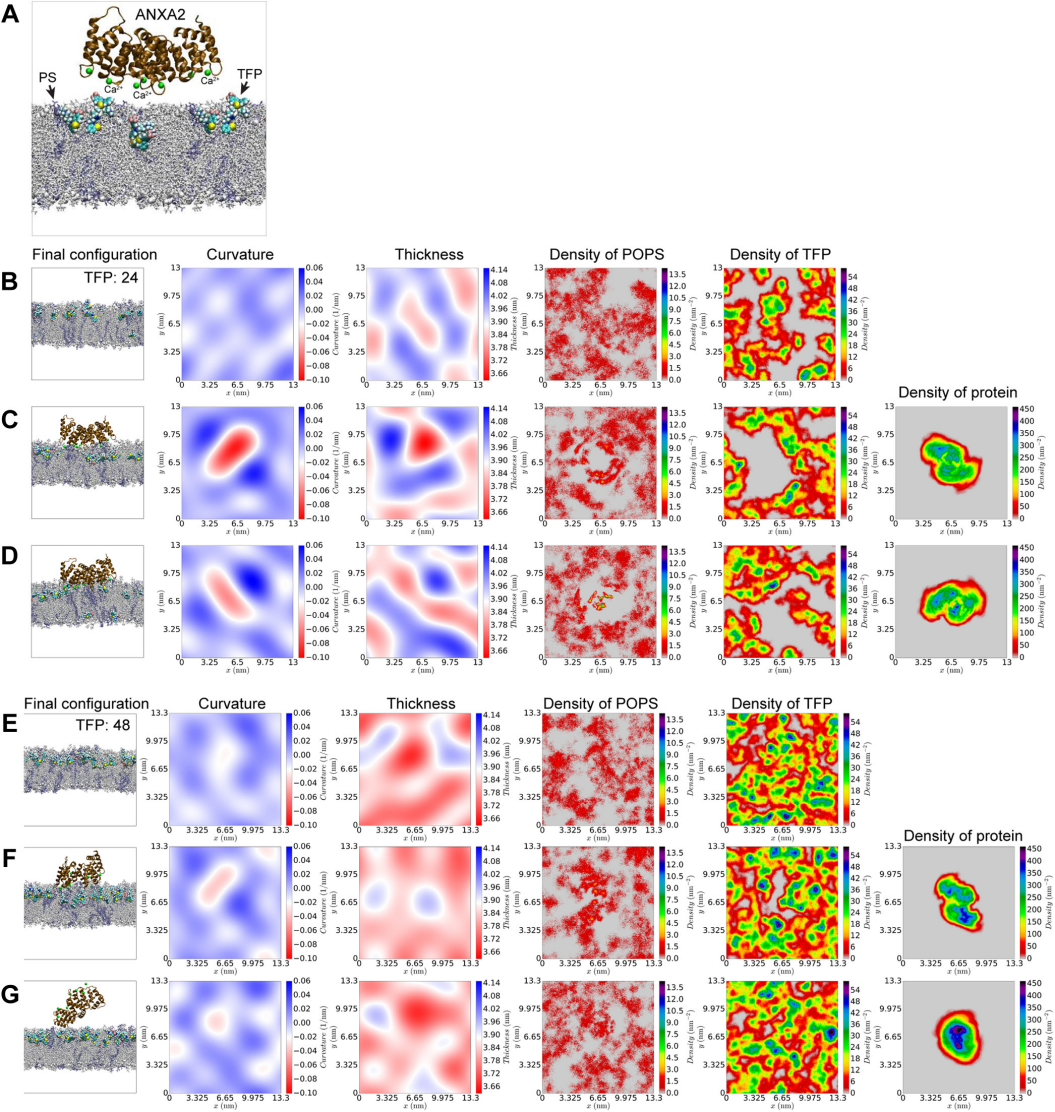
**Simulations of membrane systems with TFP ± ANXA2.***A*, membrane (20% POPS, 80% POPC) system with ANXA2 and TFP. In configuration snapshot, POPC lipids are shown in *gray* and POPS lipids are shown in *blue*. Ca^2+^ ions are shown as *green spheres*. *B* and *E*, final configuration of pure membranes and profiles of curvature, thickness, upper monolayer POPS density, and density of TFP molecules in systems with 24 and 48 molecules of TFP molecules respectively. *C* and *D*, two representative simulation replicates of systems with 24 TFP molecules and ANXA2. *F* and *G*, two representative simulation replicates of systems with 48 TFP molecules and ANXA2. See also Video S6, Figs. S3 and S4 for data from the other simulation replicates.

For molecular-level insight into how TFP-treated membranes affect ANXA2 binding and curvature induction, we performed all-atom molecular dynamics (MD) simulations of ANXA2 in proximity to a lipid bilayer, in the absence (Fig. 5) and presence of TFP in the membrane (Fig. 6). The interactions of ANXA2 protein with negatively charged phosphatidylserine lipids on the bilayer surface were mediated by Ca^2+^ ions (Fig. 5 and Video S5). As expected, in systems without TFP, ANXA2 bound to the membrane within 500 ns and induced negative curvature on the membrane (Fig. 5, *C* and *D*). The mean ANXA2-induced curvature averaged over five different replicas was 0.0112 ± 0.0001 nm^−1^ (Table S1).

When membrane-inserted TFP was included in the systems at two different densities (24 or 48 TFP molecules corresponding to TFP/lipid ratios of 4.6% and 9.2%, respectively) (Fig. 6, Figs. S3 and S4), ANXA2 could still bind the membrane but the binding was weaker (Video S6), in agreement with data from our cell studies (Fig. 2) and the supported membrane patch model (Fig. 3). Interestingly, TFP reduced the curvature footprint induced by ANXA2 protein to 0.0075 ± 0.0001 nm^−1^ and 0.0063 ± 0.0001 nm^−1^ in the simulations with 24 and 48 TFP molecules, respectively (Tables S2 and S3), representing a 1.49- and 1.78-fold decrease in induced mean curvature compared with systems without TFP.

As expected, TFP treatment altered the biophysical properties of membranes. TFP thins the membrane and increases the area per lipid (Fig. 6, *B* and *E* and Tables S1–S3). These findings from MD simulations agree with the AFM experiments on supported membranes, in that TFP decreases the membrane bilayer thickness and increases the patch area per lipid (Figs. 4 and 6) (40).

Specifically, we observe the following dose–effect trends for different biophysical membrane parameters (Tables S1–S3):

Thickness: 48TFP<24TFP<0TFP

Area: 48TFP>24TFP>0TFP

In addition, since ANXA binding to the membrane depends on anionic lipids, we used the three simulation systems to estimate the lateral diffusion constants (D_L_) for POPS lipid in the bilayer:

$${\text{D}}_{\text{L}}:0\text{TFP}=9.9\left(\pm 0.1\right)*10^{-8}{\text{cm}}^{2}/\text{s}$$

$${\text{D}}_{\text{L}}:24\text{TFP}=7.5\left(\pm 0.1\right)*10^{-8}{\text{cm}}^{2}/\text{s}$$

$${\text{D}}_{\text{L}}:48\text{TFP}=6.9\left(\pm 0.3\right)*10^{-8}{\text{cm}}^{2}/\text{s}$$

The data reveal that TFP affects the lateral diffusion of PS in the membrane, which implies that the availability of PS in the membrane is restricted. Thus, when TFP intercalates in membranes, it might weaken ANXA binding simply by limiting the diffusion of PS and hence, ANXA-PS binding opportunities.

Taken together, the data show that ANXA2, like other ANXAs, induces negative curvature on a POPC-POPS membrane. When TFP is added to the system, ANXA2 can still bind to a TFP-containing membrane, but the lateral diffusion of PS is reduced, which further weakens the binding. Moreover, ANXA2 has a reduced curvature footprint in the presence of TFP and thus, cannot shape membranes. When TFP intercalates into membrane bilayers, it thins the membrane making it more fragile, in excellent qualitative agreement with the AFM observations (Fig. 4).

## Discussion

Our results demonstrate that derivatives of phenothiazine, including TFP, sensitize cancer cells to PM injuries. As a result of compromised repair, cells are more sensitive to both chemical- and mechanical-induced PM damage. We provide evidence that TFP inhibits repair by altering the biophysical properties of the membrane, which has direct impact on the membrane-shaping capacity of ANXA proteins during repair. Further, the effect of phenothiazine drugs on cell viability upon damage may transcend to other internal membrane compartments including lysosomes (41, 42), which may further sensitize to damage-induced cell death.

The rational of phenothiazine repurposing for cancer therapy has been previously explored and TFP has shown promising anticancer effect in several cancer types (42, 43, 44, 45, 46). It was reported to suppress the invasion of metastatic cancer cell lines, by reducing angiogenesis (43), and to induce lysosome-dependent cancer cell death, through its ability to accumulate in acidic lysosomes (an ability of cationic amphiphilic compounds), thus sensitizing cancer cells to chemotherapy (42, 45). However, even though phenothiazines also impact on normal cells, the effect of phenothiazine derivatives on cancer cell membrane homeostasis has not been previously described and the observed TFP-induced PM damage opens novel avenue to target tumor cells. We hypothesise that the combination of derivatives of phenothiazine with targeted approaches that induce mechanical and/or heat-induced damage may have added anticancer therapeutic potential. Since TFP sensitizes cells to both mechanical and heat-induced membrane damage, it may prove advantageous in combination with, *e.g.*, high-intensity focused ultrasound (HIFU) therapy (47, 48). HIFU uses ultrasonic waves to penetrate soft tissue and produce physiological effects at the target in a noninvasive manner. It is still a relatively new approach, and there are many clinical programs investigating its expanded use in cancer therapy.

**Figure 7:**
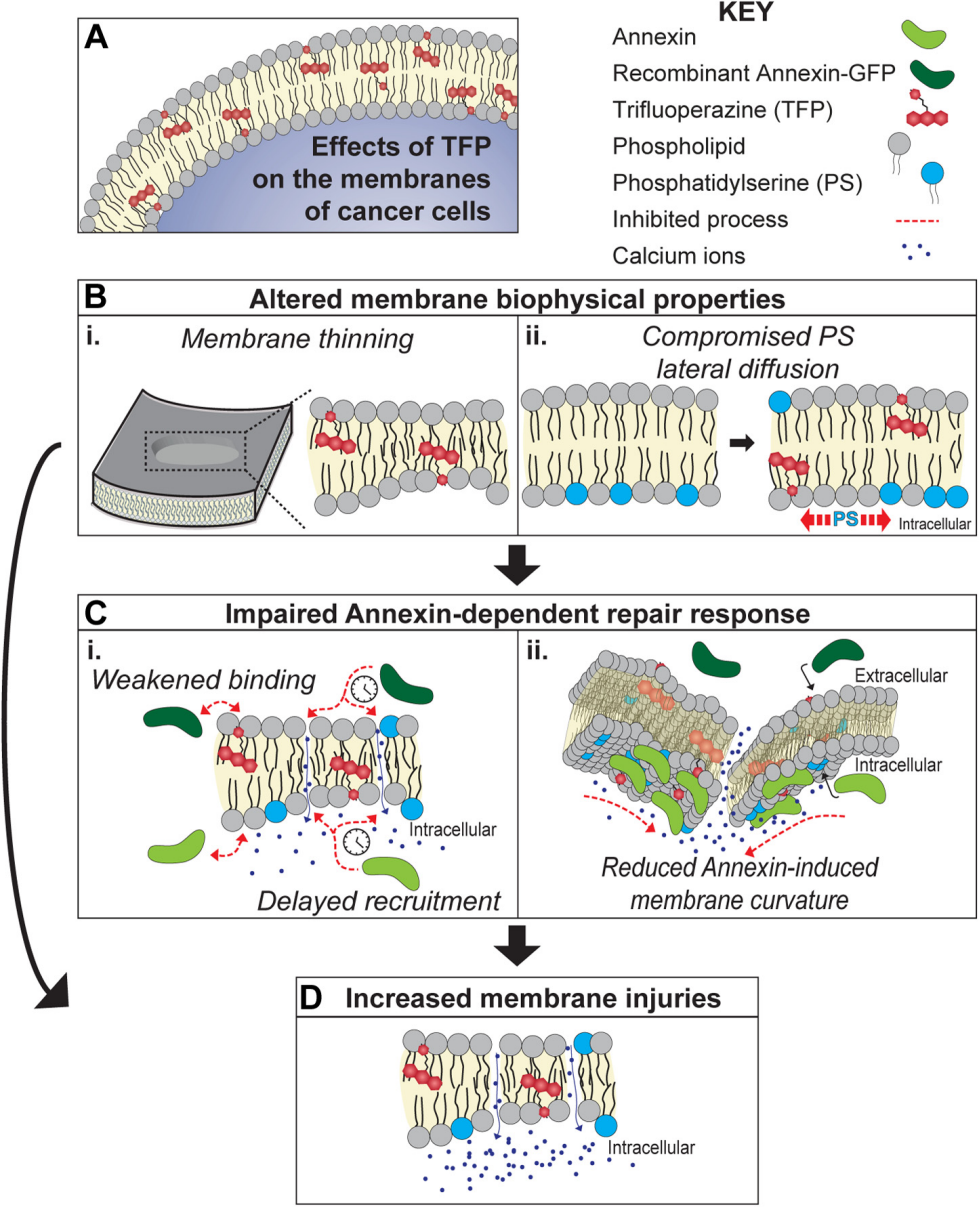
**Summary of the effects of TFP on cancer cell membranes.***A*, intercalation of TFP to the membranes of cancer cells drives a cycle that consists of detrimental changes to the biophysical properties of membranes *B*, which impair the ANXA-induced membrane repair response *C* and, in turn, results in membranes with injuries that cannot be promptly repaired *D*. The changes in membrane properties observed (including membrane thinning, *B*, i) directly contribute to an increase in membrane disruptions, as they render membranes more fragile and prone to injuries *D*. TFP-induced lateral diffusion of PS, *B*, ii, is related to the delayed recruitment of ANXAs to the membrane and the weakened membrane-ANXA interactions *C*, i, as ANXAs anchor to the membrane by binding to the negatively charged PS headgroup. This, together with the altered properties of the membrane, results in reduced ANXA-induced membrane curvature, which is key for ANXA-mediated membrane repair.

According to our data, three major aspects of TFP affect cell membrane stability and integrity (summarised in Fig. 7).

First, TFP intercalates in the lipid bilayer (Fig. 7*A*), making the PM thinner (Fig. 7*B*, i), as understood from the AFM topography measurements (Fig. 4) and MD simulations (Fig. 6). These properties render membranes prone to ruptures (26) and, in turn, more susceptible to cell death, which is supported by our cell studies (Figs. 2 and 3). This TFP-induced expansion of the molecular area of lipids has also been reported by Broniec *et al.* (49) for dipalmitoylphosphatidylserine and PS in synthetic monolayers. In fact, TFP-induced membrane permeabilization has been previously reported with higher TFP concentrations, although it was attributed to the induction of phase separation and membrane fluidisation by the drug (32). It is well established that most small amphipathic molecules, which bind to the lipid-bilayer interface and disturb lipid packing, lead to an increase in the area per lipid accompanied by a decrease in membrane thickness and lowering of lipid tail order parameters (50). The same mechanism is likely responsible for the thinning effect of TFP on the lipid bilayer. This initiates a cycle, where TFP sensitises cells to injury (Fig. 7*D*) both directly, by facilitating membrane fragility (Fig. 7*B*), and indirectly, by preventing adequate ANXA-mediated membrane repair (Fig. 7*C*).

Secondly, in the presence of TFP, ANXA recruitment to the membrane is slower and the binding of ANXAs to the membrane is weaker. This is supported by the cell experiments showing delayed translocation and accumulation of ANXA2-GFP to the damaged membrane. Furthermore, our membrane patch fluorescence experiments revealed weaker ANXA binding to patches in the presence of TFP. Although our MD trajectories are not long enough to sample several binding and unbinding events, we quantify the differences in the binding of ANXA2 to the bilayer with and without TFP in our simulations by measuring the difference in the induced curvature, accumulation of PS lipids below the protein, and differences in the properties of the membrane. Calculation of thermodynamic affinities of large proteins to membranes is beyond the scope of the current work.

Several mutually nonexclusive explanations may drive this compromised recruitment and binding. Initially, the slower diffusion coefficient of PS lipids in the presence of TFP compromises the availability of PS for ANXA binding. Moreover, being cationic compounds, phenothiazines can, to some extent, neutralize anionic lipids once intercalated in the membrane bilayer. This is supported by studies suggesting that TFP-PS interaction is mainly driven by electrostatic interactions between the TFP cation and PS headgroup anion, while the phenothiazine moiety is inserted among the acyls in the monolayer (49). Accordingly, TFP has been shown to inhibit the ability of ANXA2 tetramer to aggregate PS liposomes and fuse PS/PE liposomes with lamellar bodies (33), although TFP had no reported effect on the binding of ANXA to membranes (33, 51).

Thirdly, TFP affects cell membrane stability and integrity also by reducing the curvature footprint per single ANXA2 molecule. This combined with less and/or weaker ANXA2-membrane binding leads to even more restricted curvature induction, which in turn recruits fewer ANXA molecules including ANXA4 and ANXA5 through their curvature-sensing ability (12). Such diminished ANXA2 curvature induction was clear from the MD simulations (Fig. 6, *B*–*E*) and from the absence of curved membrane topologies in the membrane patch experiments for all ANXAs tested (ANXA1, ANXA2, and ANXA4) (Fig. 3).

The cumulative effect of all these events is likely to compromise ANXAs membrane binding. TFP changes the mean curvature by about 0.0049 nm^−1^. The mean curvature of a similar-sized protein-free membrane patch fluctuates around zero and the magnitude of the time average of mean curvature is less than 0.001 nm^−1^. Therefore, the change in curvature in the presence of TFP is significant. In addition, in combination with our other biophysical investigations, it is safe to conclude that the change may be substantial enough to prevent membrane rolling induced by membrane bending. In a previous study of Shiga toxin B-subunit (STxB), which binds to the glycolipid globotriaosylceramide (Gb3) at the plasma membrane of target cells (52), a change of mean curvature from 0.017 nm^−1^ to 0.005 nm^−1^ was sufficient to result in significant differences in large-scale membrane deformations. To this end, the mean curvature of a membrane segment under the Annexin A4 trimers has also been reported as 0.024 nm^−1^ (12).

Thus, the normal membrane-shaping function of ANXAs (3, 7, 8, 11, 22) is blocked by TFP, which results in compromised PM repair response upon injury.

Taken together, TFP initiates a detrimental cycle, where thinning of the lipid bilayer makes the membrane more prone to ruptures, which in turn are not repaired, because the ANXA-repair response is compromised. Hence, TFP and derivatives of phenothiazine appear to act as pan-ANXA inhibitors by disturbing the ANXA-mediated repair function at the PM.

## Experimental procedures

### Cell culture and treatments

HeLa cells originating from human cervix carcinoma (ATCC) and MCF-7 human breast carcinoma cells were cultured in RPMI 1640 GlutamaxTM medium (Gibco) supplemented with 6% heat-inactivated fetal bovine serum (Gibco 10270-106), 0.25% penicillin streptomycin (Gibco). NCI-H1299 originating from non-small-cell lung cancer and MDA-MB-231 cells originating from breast carcinoma were cultured in DMEM GlutamaxTM medium (Gibco) supplemented with 10% heat-inactivated fetal bovine serum (Gibco 10270-106). Cells were kept at 37 °C in a humid atmosphere of 5% CO2.

### Membrane wounding experiments

#### Laser injury

Cells were injured at 37 °C in imaging media (RPMI without phenol red with 6% FBS, 2 mM GlutaMax and 25 mM HEPES) in a small area of (2 μm diameter) by irradiating with a 355 nm UV ablation laser at ∼2.65% power setting, repetition rate of 200 Hz, pulse energy of >60 μJ, pulse length of <4 ns (Rapp OptoElectronic). The injury response was imaged with a 63x objective using Nikon confocal microscope equipped with a PerkinElmer spinning disk (for Rapp OptoElectronic pulsed UV-laser). The cells were imaged every 2 to 5 s starting before injury and continuing up to 5 min following injury. Volocity software was used to measure kinetic of repair by monitoring uptake of impermeable cellular FM1-43 dye (Life technologies) (1 mg/ml) as a change in fluorescence during the course of imaging. Volocity was equally used to analyze the translocation kinetics for ANXA2-GFP of control and TFP-treated cells and plotted over time as F/F0.

For recombinant protein studies, cells were incubated in imaging media with 20 μg/ml recombinant ANXA2 protein or Bovine Serum Albumin ± TFP for 5 min before imaging. FM 1-43 dye (2 μM) was added to the cells just prior to imaging. Images were acquired and quantitated as described above.

#### Detergent-induced injury

Cells were incubated with digitonin (Sigma-Aldrich) in different concentrations (0–20 μg/ml) for 30 min. To measure membrane integrity and cell viability, cells were incubated with 2.5 μg/ml Hoechst-33342 (Sigma-Aldrich) (Excitation 350, Emission 461) and 2 μg/ml propidium iodide (Sigma-Aldrich) (Excitation 535, Emission 617), and integrity was measured using Celigo imaging cytometer (Brooks Life Science Systems) and analyzed using Celigo Software Version 2.1.

#### Glass bead injury

Cells in suspension were mixed with 250 mg glass beads (425–600 μm Sigma-Aldrich) in Eppendorf tubes. Cells were incubated with 15 or 30 μM TFP at 37 °C for 1 h followed by vortexing for 0 to 3 min and incubation at 37 °C for 5 min to allow repair.

#### Heat-induced injury

Cells were incubated with TFP (0–30 μM) for 30 min at 37 °C followed by heat exposure on a heat block for 15 min, leading to a gradual temperature increase, reaching a final temperature of 55 °C in the cell suspension.

#### Injury by sonication

Cells in suspension were incubated with TFP at 37 °C followed by Sonication for 1 or 3 s using Bioruptor UCD-300 (Diagenode) waterbath at 4 °C with the following settings: ultrasonic wave frequency of 20 kHz, ultrasonic wave output power of 320 W. Cell death was measured by Celigo imaging cytometer.

### Plasmid construct

Cells were transfected with an expression plasmid containing human ANXA2 cDNA with a turbo-GFP C-terminal tag purchased from OriGene Technologies (#RG205081) using LipofectamineTM LTX (Thermo Fisher) according to the manufacturer’s protocol.

### Protein production

Recombinant ANXAs were produced and purified as previously described (7, 8). Briefly, ANXA cDNA constructs were subcloned into the bacterial expression vector pETM11-SUMO3 (originally from EMBL Protein Expression and Purification Core Facility) with or without C-terminally tagged superfold GFP (sfGFP) and N-terminally tagged with a His6-tag and a SUMO3 domain. BL21 (DE3) competent *Escherichia coli* cells were used as expression hosts for the production of recombinant proteins, and the expressions were induced overnight at 18 °C using Isopropyl β-D-1 thiogalactopyranoside (IPTG). Cells were harvested by centrifugation and lysed mechanically by sonication. His6-tagged proteins were purified using Immobilized Metal-Affinity Chromatography (IMAC) with Ni-NTA (nickel-nitrilotriacetic acid) resins (Qiagen). The His6-tag and SUMO3 domain were cleaved of by SUMO-Specific Protease 2 (SENP2) (200:1) followed by dialysis overnight at 4 °C. Proteins were further purified and separated using Fast Protein Liquid Chromatography (FPLC) on a Superdex 200 size-exclusion chromatography column (SuperdexTM 200, 10/300 GL, GE Healthcare Life Sciences). Protein fractions were collected and stored at −80 °C until use.

### Supported lipid bilayer membranes for membrane patch experiments

Mica substrates (Plano GmbH) were prepared by cutting thin sheets and glue to glass coverslips using silicone elastomer (MED-6215, Nusil Technology). Lipid films of PC(POPC;1-hexadecanoyl-2–(9Z-octadecenoyl)-sn-glycero-3-phosphocholine) and PS(POPS;1-hexadecanoyl-2–(9Z-octadecenoyl)-sn-glycero-3-phospho-L-serine) were prepared from a stock solution containing 10 mM total lipid (POPC, POPS, 9:1 M ratio) and 0.5% DiD-C18 probe (Thermo, Invitrogen). A 20 μl droplet of the lipid stock was applied to the mica and spun on a spincoater (KW-4A, Chemat Technology) at 3000 rpm for 40 s and placed under vacuum in a desiccator for 10 to 12 h to ensure evaporation of the solvent. The spincoated lipid film was hydrated in TRIS buffer (10 mM Tris (pH = 7.4), 140 mM NaCl, 2 mM CaCl_2_, pH = 7.4 at 55 °C for 2 h). Then the sample was gently flushed with 55 °C fresh buffer for >10 times to prepare defined secondary bilayer patches on top of a continuous primary membrane. The membranes where cooled to 22 °C and equilibrated for 1 to 2 h before further experiments. The interaction of ANXAs and TFP with membrane patches was monitored at 22 °C with time-lapse epi-fluorescence microscopy (DiD was used as membrane marker) using a Nikon TE2000 inverted microscope (x40 objective, Nikon ELWD, Plan Fluor, NA = 0.6). Fluorescence excitation was performed with a switchable Xenon lamp (PolychromeV, Till Photonics GmbH) and a dual wavelength for imaging at 640 nm (DiD) and 488 nm (GFP). ANXA proteins in a final amount of 100 pmole were added to the fluid cell from a concentrated stock with a known concentration of typically 1 mg/ml, and the sample was imaged at 3 to 10 frames per second, depending on the response speed of the ANXA protein. GFP-labeled ANXAs were imaged simultaneously to DiD by switching the wavelength of the excitation.

Quantification of lipid patch area over time was performed using ImageJ. The image sequence obtained from time-lapse microscopy was imported and “Freehand tool” was used to mark and measure membrane area. The measurement scale was set by drawing a line between two points of known distance. For experiments with TFP and ANXAs, ANXAs were added immediately after TFP-membrane intercalation was observed (t = 0). For experiments with only TFP, t = 0 marks the timepoint where TFP intercalation was observed. The membrane area measurements over time were normalized to t = 0 (7).

### Supported lipid bilayer preparation for atomic force microscopy

Phosphatidylcholine (POPC; 1-hexadecanoyl-2– (9Z-octadecenoyl)-sn-glycero-3-phosphocholine), phosphatidylserine (POPS; 1-hexadecanoyl-2– (9Z-octadecenoyl)-sn-glycero-3-phospho-L-serine) were solubilized in methanol at a ratio of POPC:POPS = 9:1 with DiD-C18 probe added at 0.5% (Thermo, Invitrogen). Solubilized mixed lipids were dried by a nitrogen flow for 30 min. Further drying was achieved by storing lipids in a vacuum chamber overnight. Dried lipids were hydrated in 10 mM TRIS buffer (2-Amino-2-(hydroxymethyl)propane-1,3-diol), 140 mM NaCl, 2 mM Ca2+, pH = 7.4 at 55 °C for 2 h and then vortexed to form multilamellar vesicles. The resulting solution was tip sonicated for 10 min at 30 W in an ice/water bath to form small unilamellar vesicles (SUVs). SUVs were deposited on freshly cleaved mica secured in an AFM fluid cell, to form a supported lipid bilayer (SLB). After 15 min, excess lipids were rinsed away with buffer. The fluid cell was then filled with buffer, sealed, and heated at 55 °C for 30 min. SLBs were then visualized with a Nikon TI2E inverted microscope and flushed with buffer, prewarmed to 55 °C, to remove any remaining excess lipid. SLBs were then allowed to cool slowly to room temperature such that large holes formed as SLBs contracted.

### Atomic force microscopy measurements of supported lipid bilayer height

AFM imaging of supported lipid bilayer membranes was performed using a Nano Wizard 4 (JPK, Bruker) operated in alternating contact mode. Ultrashort cantilevers (USC-F0.3-k0.3, Nano World) with a nominal spring constant of 0.35 Nm^−1^ and a probe radius of 10 nm (according to manufacture specifications) were used. Scanning was performed using the smallest possible contact force to minimize potential membrane deformation. Samples were imaged in 0, 7, 15, and 30 μM TFP solutions prepared in 10 mM TRIS buffer (2-Amino-2-(hydroxymethyl)propane-1,3-diol), 140 mM NaCl, 2 mM Ca^2+^, pH = 7.4, after allowing 10 min equilibration time for each concentration change. Three images at different locations on the sample were taken per condition. Processing of AFM images was done with both Gwyddion (Czech Metrology Institute) and JPK Data Processing software (JPK, Bruker). Ten lines (five pixels thick) were drawn across SLB/hole edges in SLBs in each image and viewed as cross sections for the determination of SLB height.

### MD simulations

We built three systems from human ANXA2 with seven calcium atoms bound (PDB: 2HYW) (53). The membrane is composed of 520 lipid molecules, 20% POPS, and 80% POPC, constructed using CHARMM-GUI (54, 55, 56, 57, 58, 59, 60). A large membrane patch was chosen to ensure vanishing protein-induced curvature near the box boundaries. Two of the systems also include TFP molecules bound to the membrane with different densities (24 TFP molecules in one system and 48 TFP molecules in another one). We built five replicas of each system with different initial distribution of lipids in the membrane and different initial velocity distributions. We also built three systems without protein, one without TFP molecules, one with 24 TFP molecules, and another with 48 TFP molecules. All structures with protein were immersed in a periodic box of TIP3P atomistic water (61) with ∼54k water molecules and 1.5 mM KCl salt corresponding to the physiological conditions. The three structures without protein were immersed in a periodic box of TIP3P atomistic water (61) with ∼34k water molecules and 1.5 mM KCl salt.

Simulations were performed using Gromacs2019 (62, 63) package and the CHARMM36 (64, 65) force field. The force field parameters of TFP were obtained from the www.paramchem.org. Since the structure of TFP is close to previously parameterized chlorpromazine, the penalty scores associated with the uncertainty in parameters were sufficiently low.

The systems with protein (without protein) were simulated for 500 ns (300 ns) at 310 K using the Nosé–Hoover thermostat (66). The pressure was kept constant at 1 atm using Parrinello–Rahman barostat (67, 68). The final systems with protein and without TFP molecules, with 24 and 48 TFP molecules measured 12.8 × 12.8 × 13.9 nm^3^, 13 × 13 × 13.4 nm^3^, and 13.3 × 13.3 × 13.3 nm^3^, respectively and consisted of ∼234k atoms. Initial structures for systems with protein, and with and without TFP molecules, are shown in Figures 5*A* and 6*A*.

To quantify the induced curvature, we used the last 200 ns of the simulations to find the curvature profile by fitting the phosphorus atom coordinates of the lipids in each monolayer to a Fourier series in two dimensions as it is explained in Ref (69). We also calculated the profile of the thickness of the membrane by the same method. The lateral diffusion constant was calculated using the Einstein relationship. We set the time between the reference points for the MSD calculation to 100 ps. We calculated the diffusion constant by least squares fitting a straight line through the MSD(t) from 0 to 50 ns (t is time from the reference positions).

$$\text{MSD}\left(t\right)=\left\langle {\left(r\left(t\right)-r\left(t_{0}\right)\right)}^{2}\right\rangle =4D\left(t-t_{0}\right)$$

where *D* is the diffusion coefficient, and the average is taken over all POPS lipids and different initial values of *t*_*0*_. The error estimate was made from the difference of the diffusion coefficients obtained from fits over the two halves of the fitting interval.

## Data availability

All data are contained within the article or available on request.

## Supporting information

This article contains supporting information.

## Conflict of interest

The authors declare no competing financial interests or other conflicts of interest.
