# Supplementary material for: Phenothiazines alter plasma membrane properties and sensitize cancer cells to injury by inhibiting annexin-mediated repair
Source: J Biol Chem. 2021 Jul 26;297(2):101012. doi: 10.1016/j.jbc.2021.101012 (PMC8363839; doi:10.1016/j.jbc.2021.101012)
Supplement: Supplemental Figures S1–S4 and Tables S1–S3 [file mmc1.pdf]

SUPPORTING INFORMATION

Phenothiazines sensitize cancer cells to injury by altering membrane biophysical properties and compromising annexin-mediated plasma membrane repair

Authors:

Anne Sofie Busk Heitmann[1], Ali Asghar Hakami Zanjani[2], Martin Berg Klenow[2], Anna Mularski[2], Stine Lauritzen Sønder[1], Frederik Wendelboe Lund[2], Theresa Louise Boye[1], Catarina Dias[1], Poul Martin Bendix[3], Adam Cohen Simonsen[2], Himanshu Khandelia[2], Jesper Nylandsted[1, 4]

Affiliations:

[1]Membrane Integrity, Cell Death and Metabolism, Center for Autophagy, Recycling and Disease, Danish Cancer Society Research Center, Strandboulevarden 49, DK-2100 Copenhagen, Denmark.
[2]PHYLIFE: Physical Life Science, Department of Physics, Chemistry and Pharmacy, University of Southern Denmark, Campusvej 55, DK-5230 Odense M, Denmark.
[3]Niels Bohr Institute, University of Copenhagen, Blegdamsvej 17, DK-2100 Copenhagen, Denmark.
[4]Department of Cellular and Molecular Medicine, Faculty of Health Sciences, University of Copenhagen, DK-2200 Copenhagen N, Denmark.

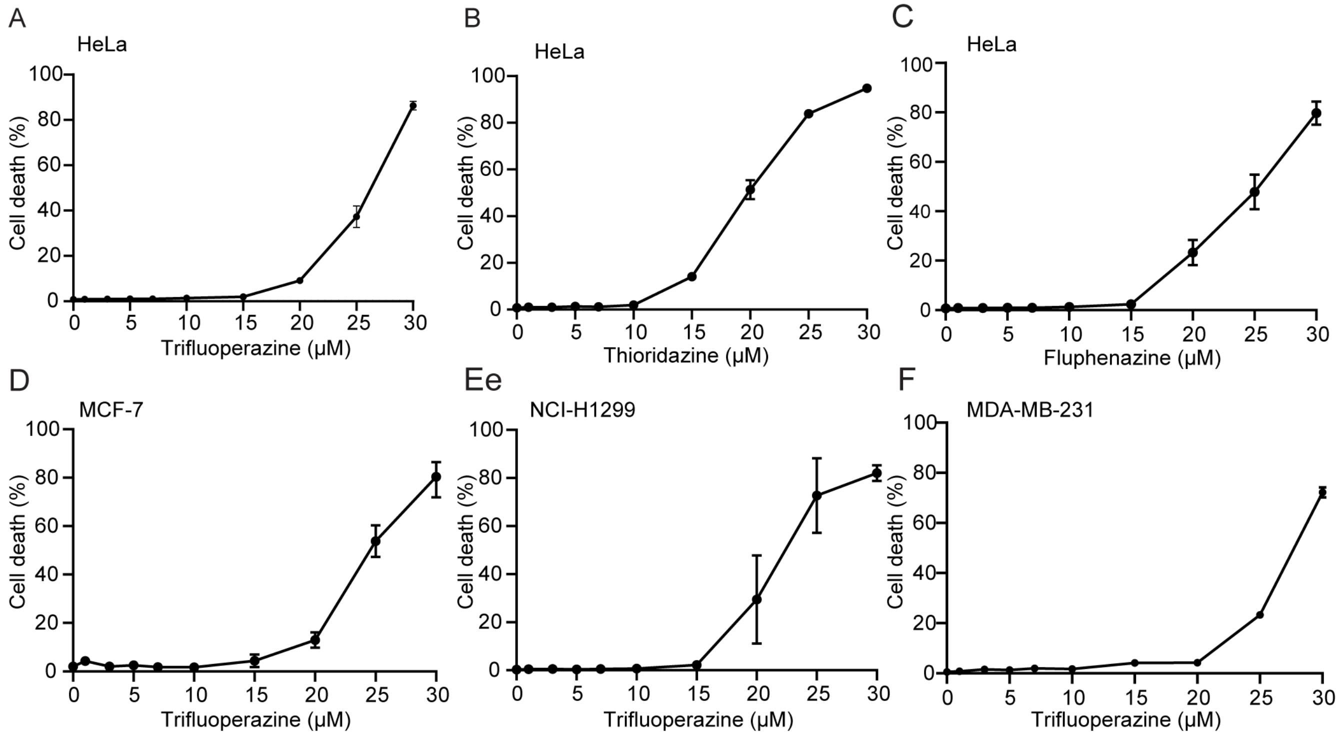

Figure S1: Effects of thioridazine, fluphenazine, and TFP on cell death after 24h.
Propidium iodide (PI) and Hoechst-33342-exclusion cell death assay of HeLa cells incubated with indicated concentrations of *A*, TFP, *B*, Thioridazine or *C*, Fluphenazine. *D-F* cell death measurements of MCF-7, NCI-H1299, and MDA-MB-231 cancer cells incubated with TFP for 24 h. Mean ±S.D of three measurements per condition.

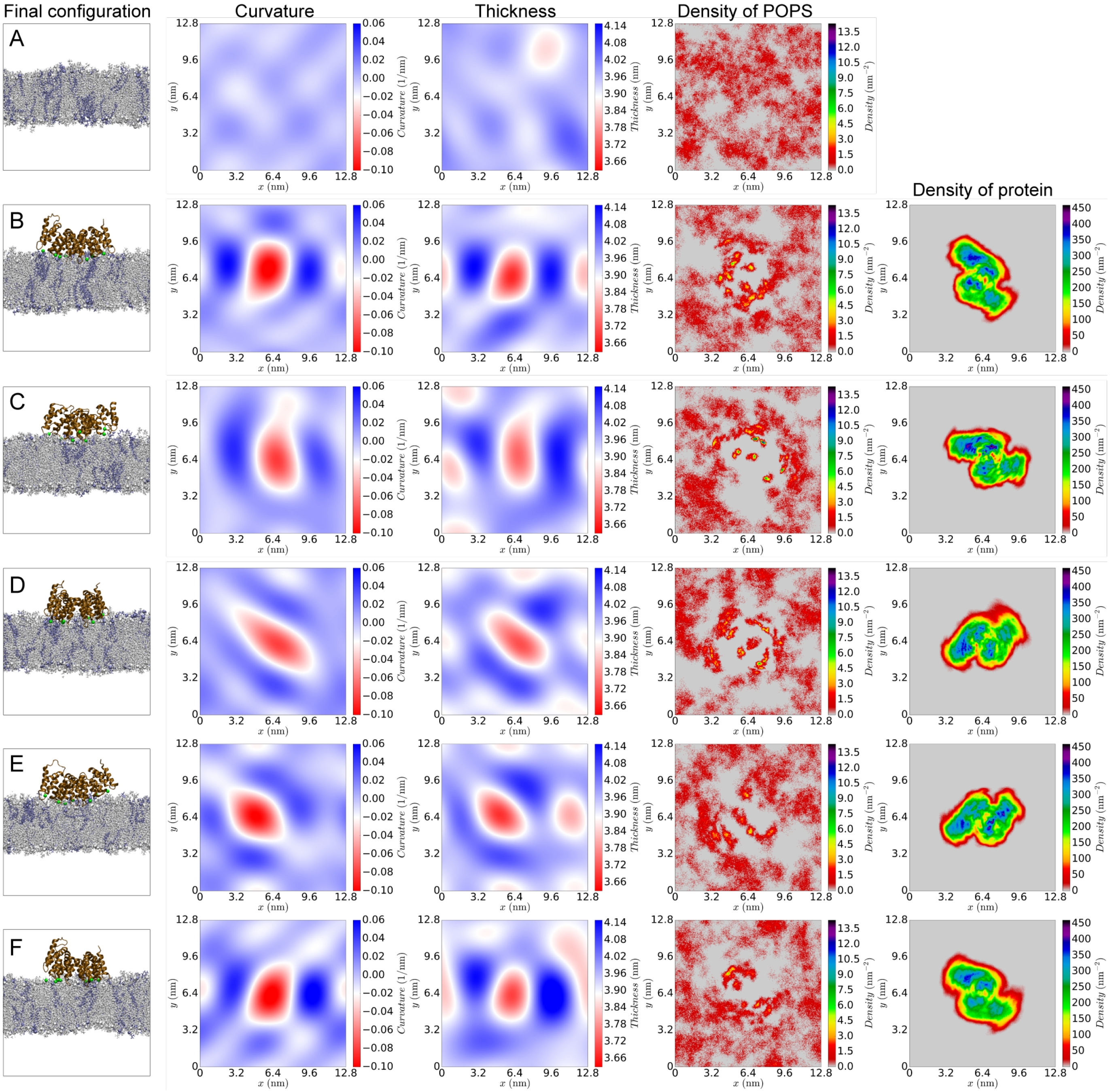

Figure S2: Final configuration and profiles of curvature, thickness, upper monolayer POPS density and density of ANXA2 protein in MD simulation systems without TFP molecules. A, Pure membrane (20% POPS, 80% POPC). *B-F*, Simulation replicates with ANXA2. In configuration snapshots, POPC lipids are shown in gray and POPS lipids are shown in blue. Ca^{2+} ions are shown as green spheres.

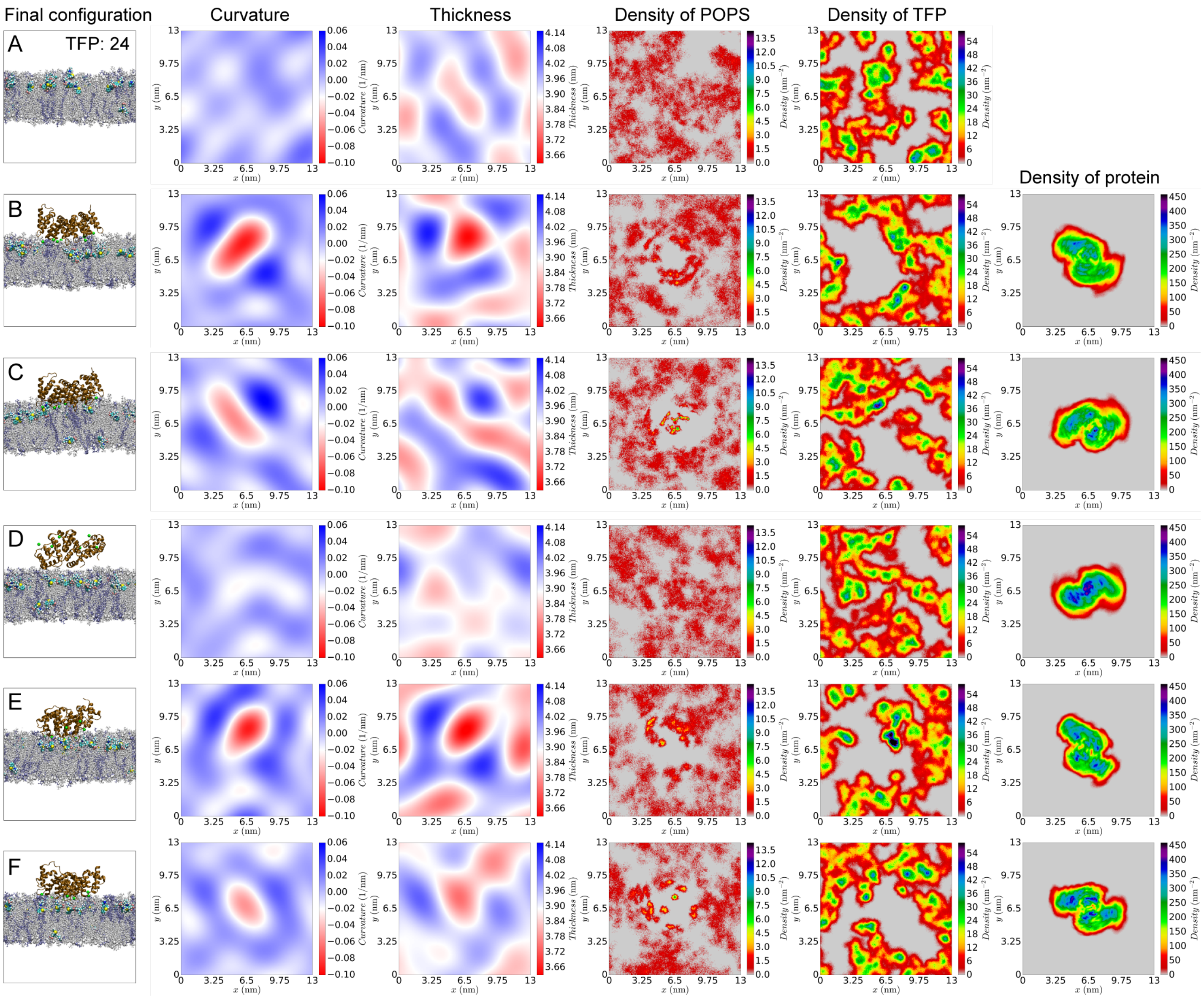

Figure S3: All atom MD simulations of membrane (20% POPS, 80% POPC) systems with 24 TFP ± ANXA2. Final configuration and profiles of curvature, thickness, upper monolayer POPS density, density of TFP molecules and density of ANXA2 protein in low-density TFP systems. *A*, Membrane with 24 TFP molecules. *B-F*, simulation replicates with TFP molecules and ANXA2. In configuration snapshots, POPC lipids are shown in gray and POPS lipids are shown in blue. Ca^{2+} ions are shown as green spheres.

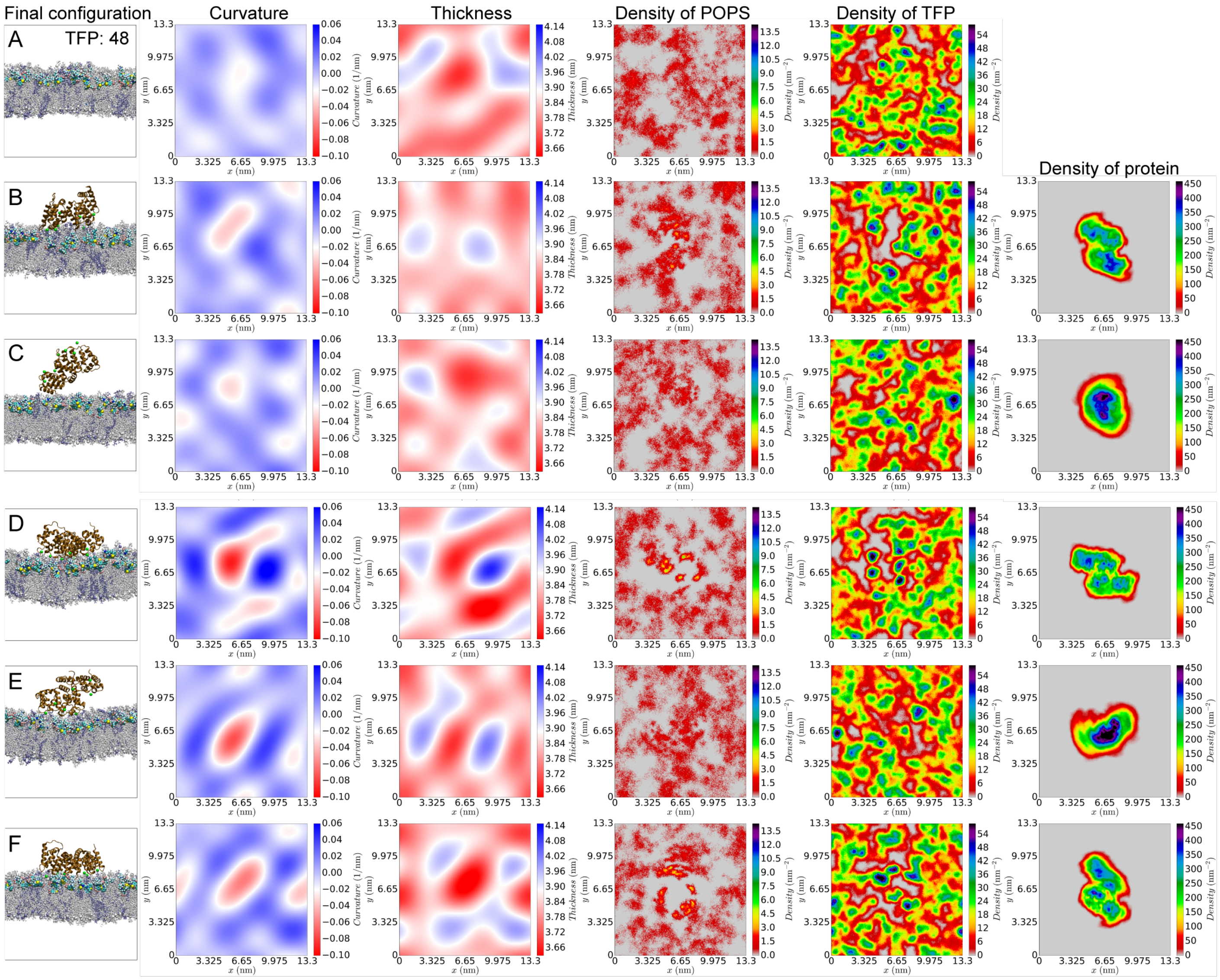

Figure S4: Simulations of membrane (20% POPS, 80% POPC) systems with TFP ± ANXA2. Final configuration and profiles of curvature, thickness, upper monolayer POPS density, density of TFP molecules and density of ANXA2 protein in high-density TFP systems. *A*, membrane with 48 TFP molecules. *B-F*, simulation replicates with TFP molecules and ANXA2. In configuration snapshots, POPC lipids are shown in gray and POPS lipids are shown in blue. Ca^{2+} ions are shown as green spheres.

Supporting Tables

0 TFP	Mean curvature (nm^{-1})	Thickness (nm)	Projected area (nm^2)
**Mem.** **a**		**3.956 ± 0.0002**	**164.597 ± 0.0366**
Mem.+A2 b	0.0105 ± 0.0003	3.951 ± 0.0004	164.969 ± 0.0380
c	0.0112 ± 0.0002	3.957 ± 0.0004	164.615 ± 0.0388
d	0.0128 ± 0.0003	3.948 ± 0.0003	164.603 ± 0.0394
e	0.0113 ± 0.0002	3.950 ± 0.0004	164.932 ± 0.0392
f	0.0101 ± 0.0002	3.955 ± 0.0004	164.715 ± 0.0369
**Avg. (b...f)**	**0.0112 ± 0.0001**	**3.952 ± 0.0002**	**164.767 ± 0.0172**

Table S1: Mean curvature under the protein, thickness and projected area of the membrane (Mem.) for systems without TFP molecules (see Fig. 5 and Fig. S2). All systems with protein ran for 500 ns and the system without protein (a) ran for 300 ns. The last 200 ns were used for calculating the values in the table.

24 TFP	Mean curvature (nm^{-1})	Thickness (nm)	Projected area (nm^2)
**Mem.+TFP** **a**		**3.915 ± 0.0003**	**169.873 ± 0.0391**
Mem.+TFP+A2 b	0.0152 ± 0.0002	3.911 ± 0.0004	170.148 ± 0.0385
c	0.0045 ± 0.0002	3.912 ± 0.0004	169.960 ± 0.0362
d	0.0050 ± 0.0002	3.908 ± 0.0002	170.236 ± 0.0383
e	0.0103 ± 0.0002	3.902 ± 0.0005	170.542 ± 0.0374
f	0.0024 ± 0.0003	3.906 ± 0.0003	170.343 ± 0.0417
**Avg. (b...f)**	**0.0075 ± 0.0001**	**3.908 ± 0.0002**	**170.246 ± 0.0172**

Table S2: Mean curvature under the protein, thickness and projected area of the membrane (Mem.) for systems with 24 TFP molecules (related to Fig. 6 and Fig. S3). All systems with protein ran for 500 ns and the system without protein (a) ran for 300 ns. The last 200 ns were used for calculating the values in the table.

48 TFP	Mean curvature (nm^{-1})	Thickness (nm)	Projected area (nm^2)
**Mem.+TFP** **a**		**3.827 ± 0.0003**	**177.771 ± 0.0430**
Mem.+TFP+A2 b	0.0032 ± 0.0002	3.836 ± 0.0002	177.138 ± 0.0383
c	0.0019 ± 0.0003	3.830 ± 0.0003	177.506 ± 0.0397
d	0.0111 ± 0.0003	3.837 ± 0.0004	177.314 ± 0.0407
e	0.0084 ± 0.0002	3.834 ± 0.0003	177.294 ± 0.0373
f	0.0071 ± 0.0003	3.836 ± 0.0004	177.252 ± 0.0391
**Avg. (b...f)**	**0.0063 ± 0.0001**	**3.835 ± 0.0001**	**177.301 ± 0.0175**

Table S3: Mean curvature under the protein, thickness and projected area of the membrane (Mem.) for systems with 48 TFP molecules (related to Fig. 6 and Fig. S4). All systems with protein ran for 500 ns and the system without protein (a) ran for 300 ns. The last 200 ns were used for calculating the values in the table.

Video Legends

Video S1: Response of membrane patch stained with DiD before and after addition of recombinant ANXA2 protein. Time: min and s. Related to Fig. 3c.

Video S2: Response of membrane patch stained with DiD before and after addition of recombinant ANXA2 protein + 15 μM TFP. Time: min and s. Related to Fig. 3c.

Video S3: Response of membrane patch stained with DiD before and after addition of recombinant ANXA4 protein. Time: min and s. Related to Fig. 3e.

Video S4: Response of membrane patch stained with DiD before and after addition of recombinant ANXA4 protein + 15 μM TFP. Time: min and s. Related to Fig. 3e.

Video S5: Representative all atom MD simulation of membrane system without TFP. Membrane (20% POPS, 80% POPC) and ANXA2 protein. POPC lipids are shown in gray and POPS lipids are shown in blue. Ca^{2+} ions are shown as green spheres. Related to Fig. 5.

Video S6: Representative all atom MD simulation of membrane system with TFP (48 molecules). Membrane (20% POPS, 80% POPC) and ANXA2 protein. POPC lipids are shown in gray and POPS lipids are shown in blue. Ca^{2+} ions are shown as green spheres. Related to Fig. 6.
